# Regional Variation in Pulse Transit Time in the Upper Limb Arteries During Hypotensive and Non-hypotensive Lower Body Negative Pressure

**DOI:** 10.7759/cureus.82752

**Published:** 2025-04-21

**Authors:** Koushik Roy, Dinu S Chandran, Kishore K Deepak

**Affiliations:** 1 Department of Physiology, All India Institute of Medical Sciences - Central Armed Police Forces Institute of Medical Sciences Center, New Delhi, IND; 2 Department of Physiology, All India Institute of Medical Sciences, New Delhi, IND; 3 Department of Biomedical Engineering, Indian Institute of Technology, New Delhi, IND

**Keywords:** arterial stiffness, lower body negative pressure (lbnp), non-invasive cuffless blood pressure (bp), pulse transit time (ptt), pulse wave velocity (pwv), sympathetic activation

## Abstract

Purpose: Pulse transit time (PTT) is crucial in developing non-invasive cuffless blood pressure (BP) measurement devices. Sympathetic activation, due to its effect on PTT, can lead to erroneous estimation of BP. Sympathetic activation might affect the PTT differentially depending on the site where PTT is measured in the upper limb. This study aimed to decipher regional variation in PTT in response to sympathetic activation in three segments of the upper limb arteries. Exposure to graded lower body negative pressure (LBNP) at hypotensive (-30 mmHg and -40 mmHg) and non-hypotensive (-10 mmHg and -20 mmHg) levels has been used to produce sympathetic activation.

Methods: This was a pilot study. Ten healthy subjects were recruited for the study, and recordings were done. Carotid, brachial, and radial pulse waveforms were recorded simultaneously by tonometry, and the finger pulse waveform was recorded by photoplethysmography (PPG). LBNP was applied at -10 mmHg, -20 mmHg, -30 mmHg, and -40 mmHg for two minutes. Carotid-brachial PTT (cbPTT), brachial-radial PTT (brPTT), and radial-finger PTT (rfPTT) were calculated.

Results: cbPTT did not show any significant change, whereas both brPTT (0.02679±0.00635 sec at baseline vs. 0.02027±0.00662 sec at hypotensive LBNP; p=0.0386) and rfPTT (0.00908±0.00350 sec at baseline vs. 0.00585±0.00211 sec at hypotensive LBNP; p=0.003) showed a significant decrease in response to hypotensive LBNP. rfPTT (0.00908±0.00350 at baseline vs. 0.00534±0.00249s at non-hypotensive LBNP; p=0.0257) also showed a significant decline in response to non-hypotensive LBNP as well.

Conclusion: The current study reveals that in upper limb arteries, PTT response to LBNP shows regional variation with an accentuation of response from proximal to distal segments.

## Introduction

Pulse transit time (PTT) is crucial in developing non-invasive cuffless blood pressure (BP) measurement devices. It is defined as the amount of time taken by the pressure wave created by left ventricular contraction to move from one arterial site to another [1]. The PTT is inversely proportional to pulse wave velocity (PWV), which increases with arterial stiffness [2]. So, a change in arterial stiffness will affect PTT. Sympathetic activation has been shown to increase arterial stiffness, thus increasing PWV. Hence, sympathetic activation might affect the PTT, which can lead to erroneous estimation of BP using PTT during sympathetic activation. During hypotensive episodes, there is reflex activation of the sympathetic system, which can affect the estimation of BP using PTT during hypotensive episodes [3].

The sympathetic system affects arterial stiffness by acting on the vascular smooth muscle cells (VSMCs) [2,4]. The amount of smooth muscle cells (SMC) in the arterial wall varies across muscular and elastic arteries. Muscular arteries are found in the periphery, whereas elastic arteries are situated near the heart [5,6]. Peripheral muscular conduit arteries, like the radial artery, have more smooth muscle than elastic material. In the terminal resistance arteries, the tunica media is composed of one or two layers of SMCs and is devoid of elastin [7]. Bjarnegård (2003) showed that the proximal brachial artery is elastic and the distal one is muscular [8]. Thus, the upper limb arteries become more muscular as we move from the center (axilla) to the periphery (hand); that is, the vascular smooth muscle content of the artery increases toward the periphery. VSMCs have been demonstrated to have α-adrenoceptors in the arterial system [9]. The role of α-adrenergic receptors in mediating sympathetic vascular transduction in the forearm of humans has been proven [10]. Bjarnegård in 2004 showed no effect of sympathetic activation on proximal elastic brachial artery beta-stiffness [11]. Thus, it can be inferred that the sympathetic system has a differential effect on the distal compared to the proximal part of the upper limb arteries. Most of the PTT measurements for BP estimation are done from the upper limb artery, usually from finger photoplethysmography (PPG) [12]. So, sympathetic activation might affect the PTT differentially depending on the site where PTT is measured in the upper limb. Sympathetic activation during lower body negative pressure (LBNP) has been shown to increase central artery carotid-femoral PWV (cfPWV) and peripheral artery carotid-brachial PWV (cbPWV) [13]. Another study found an increase in cfPWV during LBNP-induced sympathetic activation [2]. Boutouyrie (1994) reported that the cold pressor test and mental stress test, which induced sympathetic activation, decreased the compliance of the muscular radial artery [14].

The surrogate of PTT, i.e., PWV of the upper limb arteries, has been studied in larger arterial segments, e.g., carotid-brachial or carotid-radial PWV. However, the regional variation in PTT in the upper limb arteries during sympathetic activation has not been studied. The change in the upper limb arteries from elastic to muscular nature is gradual, so there might be a regional variation in the PTT. The present study aims to decipher the regional variation in PTT in the upper limb arteries by dividing it into three arterial segments, i.e., carotid-brachial, brachial-radial, and radial-finger arterial segments, during hypotensive and non-hypotensive LBNP.

The application of LBNP is one of the experimental models used to activate the sympathetic nervous system. At lower grades of LBNP, such as -10 mmHg and -20 mmHg, not much change is seen in the BP, as the amount of lower body venous pooling is minimal. BP can be quickly corrected by baroreceptors. However, at higher grades of LBNP, such as -30 mmHg and -40 mmHg, due to a higher amount of lower body venous pooling, even with baroreceptor activation, a reduction in BP can be observed. Therefore, lower grades (-10 mmHg and -20 mmHg) of LBNP are considered non-hypotensive, while higher grades (-30 mmHg and -40 mmHg) are considered hypotensive [15-18].

## Materials and methods

The study was conducted between April 2019 and February 2021 in the Space and Environment Research Facility, Department of Physiology, All India Institute of Medical Sciences, New Delhi. Healthy, young male volunteers, between 25 and 35 years of age, were recruited for the study. Subjects with a history of cardiovascular and neurological disorders, syncopal attacks, smoking, or tobacco chewing for any duration were excluded from the study in consideration of the safety of the subjects and the potential confounding effect these conditions might have on the study parameters. The study was a within-subject experimental pilot study. There was no prior study to calculate the effect size, and thus, the sample size could not be calculated. We asked subjects to volunteer, and 10 healthy subjects who fulfilled the inclusion and exclusion criteria volunteered. The subjects were informed about the experimental procedure and gave informed consent before participating in the study. Then, subjects were recruited and data were collected. The study was granted ethical clearance for research on human subjects by the Institute Ethics Committee, All India Institute of Medical Sciences, New Delhi (Ref. No.: IECPG-153/28.02.2019).

Tonometer probe

**Figure 1:**
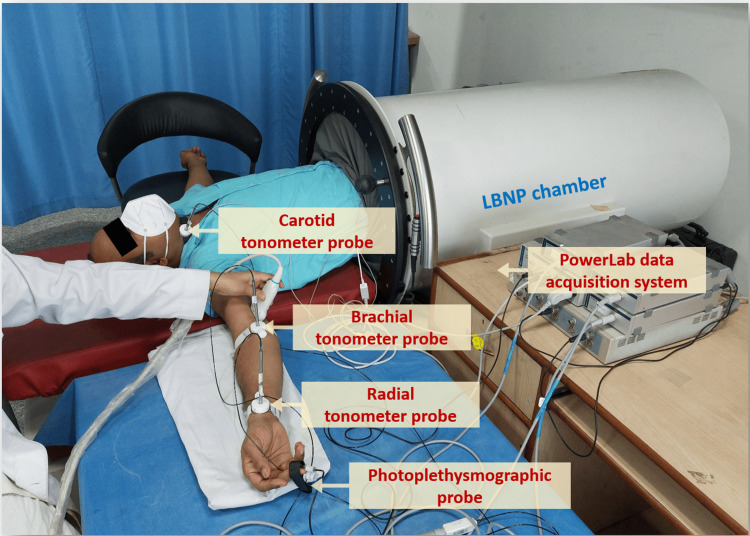
The experimental setup displaying the LBNP chamber, tonometric probes, PPG probe, and PowerLab digital data acquisition system LBNP: lower body negative pressure; PPG: photoplethysmography

Carotid, brachial, and radial pulse waveforms were obtained using high-fidelity Millar® SPT-301 non-invasive pulse wave Tonometers (Millar® Houston, Texas, USA) connected to PowerLab® 8/35 (AD Instruments, Australia) [19]. Tonometers were placed on the neck between the larynx and the anterior border of the sternocleidomastoid muscle for recording the carotid pulse waveform, at the cubital fossa for the brachial pulse waveform and at the wrist for the radial pulse waveform. Tonometers were held in position using custom-made probe holders with Velcro straps (Figure 1). Data acquisition was performed using LabChart software (Version 8.1.13; AD Instruments, Australia).

Photoplethysmographic probe

PPG signals were acquired using an infrared PPG probe [20]. The probe was placed over the middle finger of the ipsilateral hand and secured in place using velcro straps attached to the probe (Figure 1). Data acquisition was performed using LabChart software.

Acquisition of electrocardiogram signal

The electrocardiogram (ECG) was recorded using the PowerLab 15T system (AD Instruments, Australia), which has an inbuilt dual BioAmp. Disposable ECG (Ag-AgCl₂: silver-silver chloride) electrodes and shielded cables were used in the Lead II configuration. The ECG waveform was displayed on the screen using LabChart software, which was used for analysis.

Lower body negative pressure chamber

Vacusport® (Weyergans HIGH CARE® Medical, Dueren, Germany) regeneration system, an LBNP chamber, was used in this study. The LBNP device consists of a hollow cylindrical metallic chamber closed at one end, and an iris ring curtain was there at the other end, which works as a seal when closed using a rotating slider (Figure 1). The subject was asked to insert the lower half of the body, up to the iliac crests, inside the chamber while in a supine position. Sealing of the chamber was done at the level of the iliac crest. The LBNP device had a control panel outside of it that could help the investigator to set the intensity (power) and duration of negative pressure. The negative pressure generated by the device was constantly monitored and recorded using a pre-calibrated pressure transducer [17].

Method

Subjects were asked to report to the laboratory between 8:30 am and 9:30 am after being instructed to fast overnight, abstain from caffeinated beverages and alcohol for at least 18 hours, and avoid strenuous exercise for 24 hours. All recordings were undertaken in the forenoon to control for circadian influences. The total time required for recording per subject varied from 60 to 90 minutes. Recordings were conducted in a temperature-controlled (23°C-27°C) noiseless laboratory environment. The testing protocol was explained, and written informed consent was obtained from all subjects. On arrival in the laboratory, subjects were instructed to rest for five minutes. Then subjects were asked to enter the LBNP device in such a way that the lower half of the body till the iliac crest was inside the chamber, and the upper part of the body in a supine position remained outside the LBNP chamber. Subsequently, the LBNP chamber was sealed at the level of the iliac crest. The subject was asked to be in this supine position for five minutes before baseline recordings were taken. Subjects were laid supine with left and right arms abducted and extended over an adjacent tabletop at the same height as the level of the subject’s heart. Support was provided by placing rolled-up towels or bed linen beneath the abducted and extended upper limb. Resting BP was measured in the arm manually by a sphygmomanometer (until two consecutive measurements at five-minute intervals did not differ by more than 10 mm Hg; an average of two corresponding consecutive values was considered as the BP). Disposable Ag-AgCl electrodes were applied in standard bipolar limb lead II configuration for continuous acquisition of ECG. Carotid, brachial, and radial pulses were located by palpation, and tonometric probes were placed over points of palpation at the neck, cubital fossa, and wrist, respectively, and the probes were secured and fixed in position using custom-made probe holders. A PPG probe was placed over the middle finger of the right hand and held in place with velcro straps attached to the probe. All vascular parameters were recorded from the right arm of the subject (Figure 1).

Study protocol

**Figure 2:**
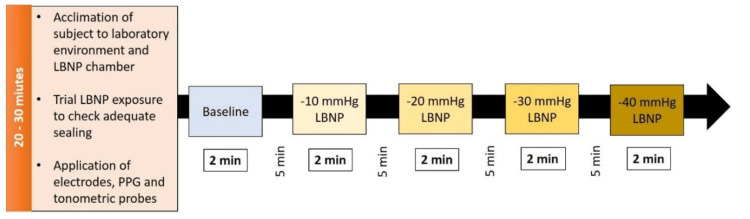
The experimental protocol along with the magnitudes and durations of LBNP used PPG: Photoplethysmography; LBNP: lower body negative pressure

The subjects were asked to report in the lab in the morning after an overnight fast (six to eight hours). After rest, baseline parameters were recorded. Following the baseline recording, LBNP was applied at -10 mmHg, -20 mmHg, -30 mmHg, and -40 mmHg pressures, each for two minutes duration. The order of application of LBNP was randomized across the study subjects. LBNP levels were randomized manually, and simple randomization was done. Acquisition of all physiological signals was continued throughout the period of application of LBNP and subsequent periods of recovery. The recovery period lasted for five minutes after the application and termination of each LBNP pressure (Figure 2).

Data analysis

**Figure 3:**
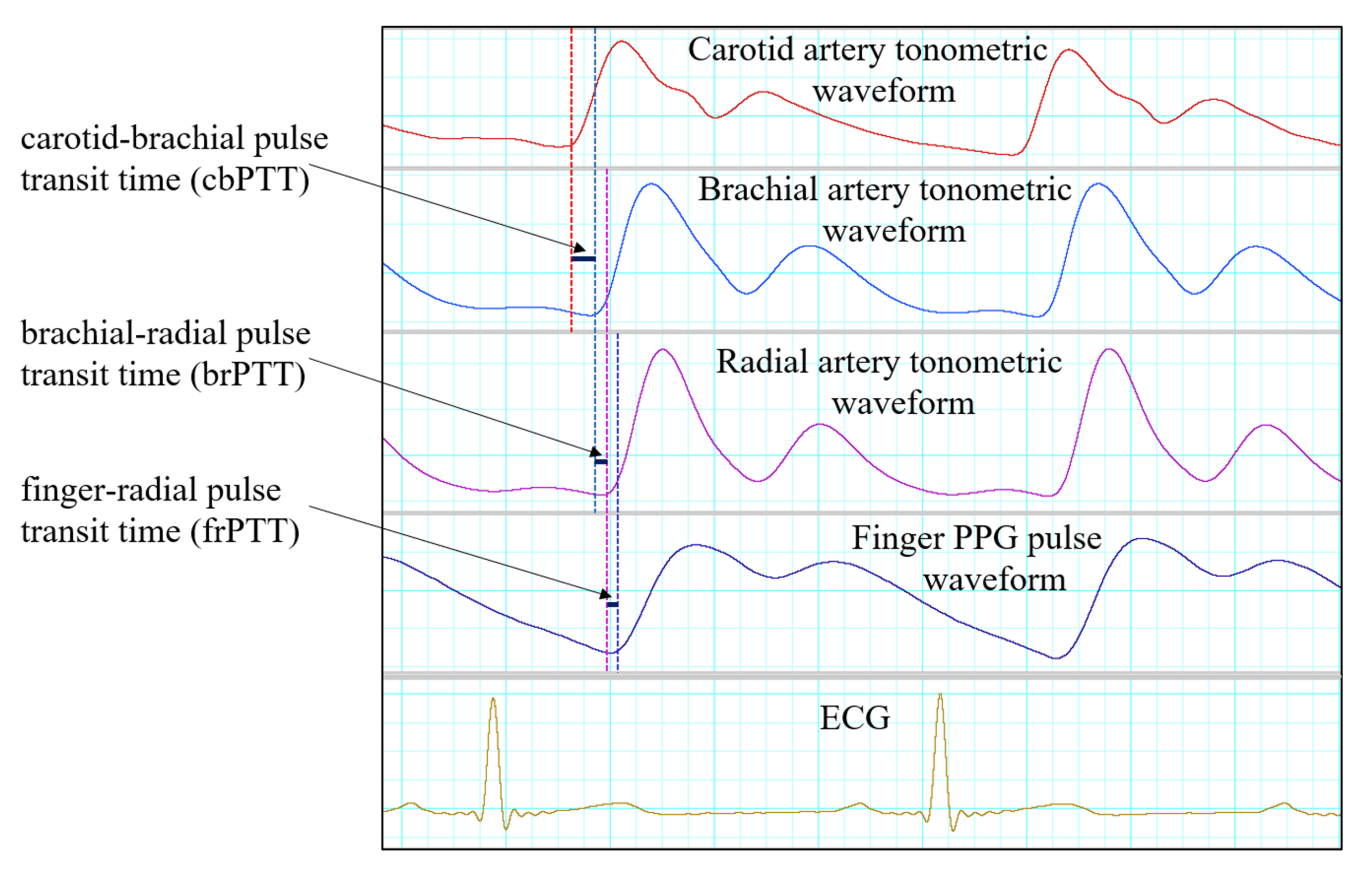
Demonstrating the method of analyzing tonometric and PPG signals to calculate different PTT ECG: Electrocardiogram; PPG: photoplethysmography; PPT: pulse transit times

Carotid, brachial, and radial tonometric pulse waveforms were filtered through a bandpass filter of 0.5 Hz-35 Hz, following which they were analyzed using the peak analysis module of LabChart software. PPG signals were analyzed for the AC component. For extraction of the AC component, PPG signals were filtered through a bandpass filter of 0.5 Hz-35 Hz and analyzed in the peak analysis module of LabChart software. Identification of the “foot” of each waveform was based on the zero-crossing in the first derivative of each waveform using the peak analysis module of LabChart software. From the peak analysis module, the start time (Tstart) of the carotid, brachial, radial, and PPG AC waveforms was extracted (Figure 3).

The time lag between the foot of carotid (carotid Tstart) and brachial (brachial Tstart) waveforms was used to calculate carotid-brachial pulse transit time (cbPTT). The time lag between the foot of brachial (brachial Tstart) and radial (radial Tstart) waveforms was used to calculate brachial-radial pulse transit time (brPTT). The time lag between the foot of radial (radial Tstart) and PPG AC (PPG AC Tstart) waveforms was used to calculate finger-radial pulse transit time (rfPTT) [21]. Acquired beat-to-beat data of all the signals were averaged in 30s blocks for the baseline and during the phase of LBNP. The 30s block during the last two minutes of LBNP was selected.

Statistical analysis

Data for each parameter was tested for normality of distribution using standard normality tests. Mixed model analysis followed by Tukey’s multiple comparison tests was used to analyze data. A mixed model analysis was done as there were some missing values. Parametric tests were applied to the data that followed a normal distribution. Since the original data of all parameters were normally distributed, descriptive statistics are expressed as mean with standard deviation (Mean±SD) throughout the study. The acceptable level of alpha error was kept at 0.05. All analyses were done using GraphPad Prism Ver 8.0.2 (GraphPad Software, San Diego, USA).

## Results

Ten male volunteers who met the inclusion and exclusion criteria were recruited, and recordings were made. Among those, one recording was excluded due to movement artifacts in the tonometric signal during the course of the LBNP application. The final analysis was done on recordings from nine subjects. Subjects had an average age of 28.11±2.92 years. The average BP of the subjects was as follows: systolic BP 116.22±4.66 mmHg and diastolic BP 78.33±6.38 mmHg (Table 1).

**Table 1:** Subject characteristics: age, systolic blood pressure, and diastolic blood pressure SD: standard deviation

Characteristics	Mean±SD
Age (years)	28.11±2.92
Systolic blood pressure (mmHg)	116.22±4.66
Diastolic blood pressure (mmHg)	78.33±6.38

Carotid-brachial pulse transit time

**Figure 4:**
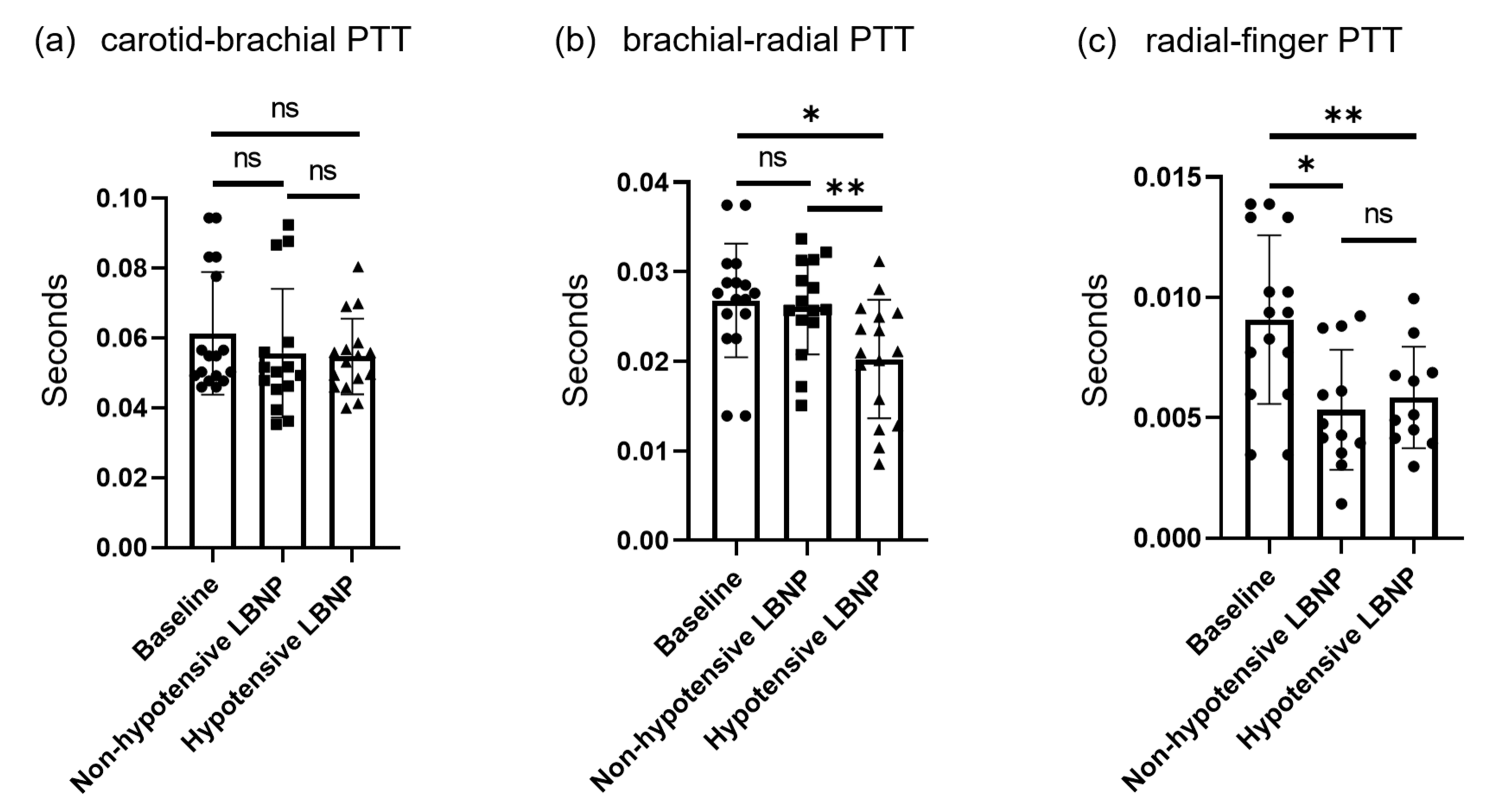
Comparison of (a) cbPTT, (b) brPTT, and (c) rfPTT between baseline (no LBNP), non-hypotensive LBNP (-10 mmHg and -20 mmHg LBNP), and hypotensive LBNP (-30 mmHg and -40 mmHg LBNP). PTTs are shown in seconds ^*^0.01<p<0.05, ^**^0.001<p<0.01 cbPTT: carotid-brachial PTT; brPTT: brachial-radial PTT; rfPTT: radial-finger PTT; LBNP: lower body negative pressure; PTT: pulse transit time; ns: not-significant

No significant difference was found in the cbPTT between baseline, non-hypotensive LBNP, and hypotensive LBNP (Figure 4(a)) (Table 2).

**Table 2:** Values of different PTTs in seconds over varying LBNP Values are expressed as mean±SD; p-values are as follows: ^^^0.0386 vs. baseline, ^#^0.0053 vs. non-hypotensive LBNP,^ $^0.0257 vs. baseline, ^γ^0.003 vs. baseline, ^a^0.4967 vs. baseline, ^b^0.1440 vs. baseline, ^c^0.9313 vs. non-hypotensive LBNP, ^d^0.9255 vs. baseline, ^e^0.6845 vs. non-hypotensive LBNP LBNP: lower body negative pressure; PTT: pulse transit time; cbPTT: carotid-brachial PTT; brPTT: brachial-radial PTT; rfPTT: radial-finger PTT

Parameters	Baseline	Non-hypotensive LBNP	Hypotensive LBNP
cbPTT	0.06131±0.01752	0.05566±0.01842^a^	0.05468±0.01084^b,c^
brPTT	0.02679±0.00635	0.02611±0.00533^d^	0.02027±0.00662^^,#^
rfPTT	0.00908±0.00350	0.00534±0.00249^$^	0.00585±0.00211^γ,^^e^

Brachial-radial pulse transit time

No significant difference was found in brPTT between baseline and non-hypotensive LBNP. However, there was a significant decrease of brPTT in hypotensive LBNP as compared to baseline. Also, there was a significant decrease in brPTT in hypotensive LBNP as compared to non-hypotensive LBNP (Figure 4(b)) (Table 2).

Radial-finger pulse transit time

There was a significant decrease in rfPTT in non-hypotensive LBNP as compared to baseline and also in hypotensive LBNP as compared to baseline. No significant difference was found in rfPTT between non-hypotensive LBNP and hypotensive LBNP (Figure 4(c)) (Table 2).

## Discussion

The present study found regional variation in PTT in the upper limb during hypotensive and non-hypotensive LBNP. In our study, even higher LBNP (hypotensive LBNP) did not affect PTT of the proximal carotid-brachial arterial segment; only a higher level of LBNP (hypotensive LBNP) could decrease PTT of the middle brachial-radial arterial segment. However, in the distal radial-finger arterial segment, even lower levels of LBNP (non-hypotensive LBNP) decreased the PTT.

At resting conditions, due to higher elastin content in central elastic arteries, elastin primarily takes the load-bearing role. However, in the peripheral muscular arteries, collagen content is relatively higher, so load-bearing is done mainly by collagen. These lead to a stiffer peripheral muscular artery than the central elastic artery [22-24]. However, at higher BP, the load-bearing of the arterial wall is done mainly by collagen, compared to lower BP, where the load-bearing of the arterial wall is done by elastin [25]. Thus, arteries become stiffer during higher BP compared to more compliant arteries during lower BP. In hypotensive conditions, in central elastic arteries, more elastin will be bearing the load in addition to the already recruited elastin, thus leading to a more compliant artery. Also, in the peripheral muscular artery during hypotensive conditions, the load-bearing role will shift from collagen to elastin; thus, the artery will become compliant.

Sympathetic activation causes contraction of VSMC via alpha-adrenoceptors [4,9]. During hypotensive LBNP, lower BP will lead to shifting of load-bearing to elastin, but there is also sympathetic activation, which will lead to contraction of VSMC, thus shifting load-bearing to VSMC from elastin, making the artery stiffer [26]. So, the VSMC content of the arterial wall will control the stiffness during sympathetic activation.

In the distal (radial-finger segment) upper limb artery, a significant decrease of rfPTT was observed in both non-hypotensive LBNP and hypotensive LBNP, as compared to baseline. However, no significant difference was found in rfPTT between non-hypotensive LBNP and hypotensive LBNP. During non-hypotensive LBNP, there is sympathetic activation, leading to VSMC contraction, thus reducing rfPTT. Another factor that could contribute to the shaping of the stiffness of the distal arteries is myogenic response [27]. However, the myogenic response will not change due to no change in BP in non-hypotensive LBNP. But in hypotensive LBNP, due to the myogenic response, there will be relaxation of VSMCs, which would counteract the effect of higher sympathetic activation on VSMCs, and thus no further reduction of rfPTT is seen in hypotensive LBNP compared to non-hypotensive LBNP.

In the middle (brachial-radial segment) upper limb artery, there was no change in brPTT between control and non-hypotensive LBNP. However, a significant decrease of brPTT was seen in hypotensive LBNP (vs. control), and a significant decrease of brPTT was also seen in hypotensive LBNP (vs. non-hypotensive LBNP). During non-hypotensive LBNP, there is sympathetic activation, leading to VSMC contraction, but no change is seen in brPTT. This might be due to the fact that the relative proportion of VSMCs is lower in the middle upper limb artery compared to the distal segment, thus reducing the effect of VSMC contraction on the stiffness of the middle upper limb artery. However, higher sympathetic activity in hypotensive LBNP compared to both baseline and non-hypotensive LBNP leads to a higher increase in stiffness of the brachial-radial segment of the upper limb artery, thus causing a decrease in brPTT [17]. The myogenic response is most prominent in the resistance arteries [27,28]. The distal upper limb arteries are mainly resistance arteries, and the middle upper limb arteries are mainly feed arteries, so the myogenic response will have less effect on the arterial stiffness of the middle upper limb arteries compared to the distal upper limb arteries [7]. So, in the arterial stiffness of the middle segment of upper limb arteries, sympathetic activation had a greater effect compared to the myogenic response.

In the proximal (carotid-brachial segment) upper limb artery, we found no change in cbPTT between baseline, non-hypotensive LBNP, and hypotensive LBNP. So, sympathetic activation by LBNP had no effect on the change of proximal brachial artery stiffness, which is elastic in nature and with relatively less vascular smooth muscle. Bjarnegard et al., 2003 and 2004, found that proximal elastic brachial artery stiffness did not change by LBNP-induced sympathetic activation, similar to the present study [8,11]. However, Holwerda 2019 reported an increase in carotid-brachial arterial stiffness at -30 mmHg LBNP but not at -15 mmHg LBNP, in contrast with the present study [13].

To our knowledge, this is the first study to record PTT across contiguous segments of the upper limb arteries and demonstrate its changes with hypotensive and non-hypotensive LBNP application. We used the PPG technique to record the finger pulse waveform, as using tonometry to do so was not feasible. PPG detects changes in blood volume, whereas tonometry detects pressure changes in arteries. Additionally, PPG signals are prone to distortion by sympathetic activity-mediated vasoconstriction [20]. This is one of the potential limitations of the present study, which might reduce the accuracy of the determination of rfPTT. We had applied LBNP to cause reflex activation of the sympathetic system but did not record the muscle sympathetic nerve activity (MSNA) during LBNP application. This is another limitation of this study. We have done the study on healthy young males, and the sample size is small. So, there is limited generalizability to the general population.

## Conclusions

In conclusion, the present study found regional variation in PTT in the upper limb arteries during hypotensive and non-hypotensive LBNP. We observed a differential behavior of the upper limb arteries as they transition from an elastic nature proximally to a muscular composition distally, during LBNP-induced sympathetic activation. There is a gradual change in PTT from the proximal toward the distal segment of the upper limb arteries. No change in PTT was observed in the proximal arterial segment, even with a higher level of LBNP (hypotensive LBNP). As we move distally to the middle arterial segment, a decrease in PTT is seen only with a higher level of LBNP (hypotensive LBNP). In the distal arterial segment, even with a lower level of LBNP (non-hypotensive LBNP), PTT was found to be decreased.
